# Clinician Perspectives on Unmet Needs for Mobile Technology Among Hospitalists: Workflow Analysis Based on Semistructured Interviews

**DOI:** 10.2196/28783

**Published:** 2022-01-04

**Authors:** April Savoy, Jason J Saleem, Barry C Barker, Himalaya Patel, Areeba Kara

**Affiliations:** 1 Center for Health Information and Communication Health Services Research and Development Service Richard L Roudebush Veterans Affairs Medical Center Indianapolis, IN United States; 2 Purdue School of Engineering and Technology Indiana University-Purdue University Indianapolis Indianapolis, IN United States; 3 Center for Health Services Research Regenstrief Institute, Inc Indianapolis, IN United States; 4 Department of Industrial Engineering James Breckenridge Speed School of Engineering University of Louisville Louisville, KY United States; 5 Indiana University Health Physicians Indianapolis, IN United States; 6 Indiana University School of Medicine Indianapolis, IN United States

**Keywords:** electronic health records, hospital medicine, user-computer interface, human-computer interaction, usability, mental workload, workflow analysis

## Abstract

**Background:**

The hospitalist workday is cognitively demanding and dominated by activities away from patients’ bedsides. Although mobile technologies are offered as solutions, clinicians report lower expectations of mobile technology after actual use.

**Objective:**

The purpose of this study is to better understand opportunities for integrating mobile technology and apps into hospitalists’ workflows. We aim to identify difficult tasks and contextual factors that introduce inefficiencies and characterize hospitalists’ perspectives on mobile technology and apps.

**Methods:**

We conducted a workflow analysis based on semistructured interviews. At a Midwestern US medical center, we recruited physicians and nurse practitioners from hospitalist and inpatient teaching teams and internal medicine residents. Interviews focused on tasks perceived as frequent, redundant, and difficult. Additionally, participants were asked to describe opportunities for mobile technology interventions. We analyzed contributing factors, impacted workflows, and mobile app ideas.

**Results:**

Over 3 months, we interviewed 12 hospitalists. Participants collectively identified chart reviews, orders, and documentation as the most frequent, redundant, and difficult tasks. Based on those tasks, the intake, discharge, and rounding workflows were characterized as difficult and inefficient. The difficulty was associated with a lack of access to electronic health records at the bedside. Contributing factors for inefficiencies were poor usability and inconsistent availability of health information technology combined with organizational policies. Participants thought mobile apps designed to improve team communications would be most beneficial. Based on our analysis, mobile apps focused on data entry and presentation supporting specific tasks should also be prioritized.

**Conclusions:**

Based on our results, there are prioritized opportunities for mobile technology to decrease difficulty and increase the efficiency of hospitalists’ workflows. Mobile technology and task-specific mobile apps with enhanced usability could decrease overreliance on hospitalists’ memory and fragmentation of clinical tasks across locations. This study informs the design and implementation processes of future health information technologies to improve continuity in hospital-based medicine.

## Introduction

Electronic health record (EHR) systems aid documentation, information retrieval, and order creation. However, their lack of portability hampers effective support of communication between health care professionals and optimal access to patient information [1-3]. Such deficiencies contribute to task redundancies, constrain medical decisions at the point of care, and create inefficiencies that detract from valuable clinician-patient interactions [4-6]. These deficits are perhaps most impactful for hospitalists, a medical subspecialty focused on inpatient needs [7,8]. Multiple factors, including high patient acuity, ineffective health information technology (IT), hospital layouts, organizational policies, and interruptions, make hospitalists’ workflow cognitively demanding and dominated by activities away from the patient’s bedside (indirect care [9-15]).

As smartphones and tablets (mobile technology) became ubiquitous, they were proposed as one way to improve health IT. Physicians in emergency departments anticipated that these devices would improve workflow and physician-patient interactions [16], and in 2012, 87% of physicians were using smartphones and tablets in the workplace [17]. However, users in health care settings report lower expectations of mobile devices after actual use [17-19]. Most studies report experiences of physicians in training or those working in emergency departments. Less is known about the perceptions of hospitalists or their unique needs [20,21].

To improve care for Veteran patients, the US Department of Veterans Affairs (VA) Mobile Health Provider Program was launched in 2014. Through this program, over 12,000 iPads have been distributed at more than 60 VA sites. The program used a multiphase implementation strategy, focused on infrastructure updates, secure access to native mobile apps, and development of VA provider apps. However, adoption and use of the iPads and mobile apps among hospitalists has been low [22]. Our objective was to describe the needs and opportunities for mobile technology during the hospitalist workday. To characterize mobile technology that can synergistically support the workflow of hospitalists, we interviewed hospitalists to gain their perspectives on integrating mobile apps.

## Methods

### Overview

We conducted semistructured interviews guided by the Systems Engineering Initiative for Patient Safety (SEIPS) framework [5,23]. This framework, consisting of five factors (people, environment, tasks, tools, and organization) and their interactions, can be used to describe how health care providers’ work systems impact workflows and outcomes [5]. Our interviews focused on tasks from multiple workflows to obtain in-depth information about related frequencies, redundancies, difficulties, and mobile apps. Our analysis aimed to characterize contributing work system factors, multiple impacted workflows, and participants’ ideas for mobile app interventions.

### Participants and Setting

The study was conducted at a 200-bed urban teaching hospital operated by VA in Indiana. This hospital offered iPad tablets and introductory training to its health care providers. We sought approximately 12 participants to increase the likelihood of thematic saturation [24,25]. We sought participants who practiced according to the hospitalist model of care because they may face overlapping workflow challenges. Physicians and nurse practitioners from hospitalist and inpatient teaching teams and second- or third-year internal medicine residents were eligible to participate [26,27]. Eligible participants were contacted via email, and a nonfiction book was offered for participation. This study was approved and overseen by the Institutional Review Board at Indiana University (#1608865326) and the Research and Development committee at Richard L. Roudebush VA Medical Center.

### Semistructured Interviews

#### Data Collection

Semistructured interviews were designed as 45-minute sessions (Multimedia Appendix 1). Participants were asked to describe their primary roles and information-intensive tasks. They were then asked to identify tasks that were frequent, redundant, and difficult, and to explain their choices [28,29]. Definitions were reviewed with participants as follows:

- Information-intensive tasks: require reading, writing, or sharing information (eg, chart review)
- Frequent: performed often or for each patient (eg, looking up patients’ contact information or reviewing discharge summaries)
- Redundant: done repeatedly that should only be done once or not at all (eg, repetitious log-ins or clicks to access required information)
- Difficult: require uninterrupted time and attention (eg, reviewing labs or determining trends in vitals)

Participants were asked to describe the context of each task with a focus on work system factors [5]. Interviewers diagrammed discussions as participants spoke. Participants completed a demographic survey, including reporting use of self- and work-furnished mobile technology. Demographics and field notes were collected on paper and scanned. Interviews were audio recorded and transcribed.

#### Workflow Analysis

For each task identified as frequent, redundant, or difficult, we open-coded participants’ responses and organized these codes within the five work system factors. Next, we analyzed the impact those tasks had on workflows [30,31]. Lastly, we analyzed participants’ responses to mobile technology to identify and describe types of potential mobile app interventions.

Using a hybrid deductive-inductive approach, we iteratively developed a code book with sections and codes to aid each type of analysis [32]. We used a deductive approach to identify relevant work system factors and an inductive approach to describe workflow effects and potential mobile app interventions. One analyst created the preliminary code book based on the SEIPS work system factors and open coding of three transcripts. Using this preliminary code book, four additional analysts reviewed another set of three transcripts. The team discussed and refined codes. With the revised code book, four analysts worked in pairs to code the remaining transcripts, which were randomly assigned. After coding each transcript independently, coding partners reviewed transcripts line by line, resolving discrepancies through consensus meetings. If new codes emerged during coding, they were retroactively applied to previously coded transcripts. Each analyst wrote memos for prominent codes; then, analyst pairs conducted consensus meetings. After these meetings, an analyst selected the most frequent open codes, linked the most frequently co-occurring codes for each, and prepared a narrative summary with supporting quotations. Coding, memo writing, and content analysis were performed using Excel (Version 2016, Microsoft Corporation).

## Results

### Participants

Over 3 months, we interviewed 12 participants: 9 staff physicians, 1 resident physician, and 2 nurse practitioners. Including residency, experience ranged from 0.9 to 37 years (mean 11.7, median 8.5); experience in the present organization was similar (0.5-37 years; mean 10.8, median 8.5). A total of 11 (92%) reported using mobile technology at work, including both personally owned and work-furnished devices. Only 3 (25%) reported using iPad tablets at work. Nurse practitioners worked on the hospitalist team, while staff physicians rotated between the nonteaching hospitalist team and inpatient teaching teams. Patient load was estimated as ranging from an average of 10 to 15 patients per day.

In the following section, we first present participants’ perceptions of specific tasks that were perceived as frequent, redundant, and difficult. Next, we describe the workflows that were perceived to be most impacted by these tasks. Finally, we present participants’ perceptions of mobile technology and potential mobile app interventions.

### Frequent, Redundant, and Difficult Tasks

Chart reviews, orders, and documentation were identified as the most frequent, redundant, and difficult tasks.

#### Chart Reviews

Participants described that chart reviews (re)established the patient’s trajectory, which included viewing patient history, recent notes, laboratory results, and vitals. Participants reported conducting a summary review for every patient throughout the day to monitor progress, orders, procedures, and test results, and estimated spending 30 minutes to review the chart of a new patient. Some participants noted that their initial reviews were completed at the beginning of the day or before their shift. This need for on-demand continuity was contrasted with fragmentation of records in EHRs and the multistep methods for accessing them. Participants characterized chart review as redundant because of intermittent updates without notification, resulting in checking either too little or too often. Information copied in workrooms and carried to patients could be outdated upon reaching patients, or effort could be wasted looking for information that had not yet arrived.

#### Orders

Participants described writing orders multiple times a day using computers. Orders included lab tests, consultations, and prescriptions. The institution currently requires electronic entry of all orders. Participants described the lack of (bedside) computers, not necessarily the need for complex thought, was what made ordering difficult and inefficient. Perception of ordering was also negatively affected by poor EHR usability. The organization of orders in the EHR was thought to be unclear, decreasing the discoverability of specific orders. Participants gave examples of order forms for similar procedures that were found in different branches of the menu. This poor organization of order forms was described as increasing difficulty by limiting application of knowledge between orders—finding and writing one type of order did not necessarily make it easier to find or write other types.

#### Documentation

Documentation was reported as one of the most labor- and time-intensive tasks. It included documenting a variety of information, including histories and physicals, visit notes, daily note, and discharge summaries. As with chart review, fragmentation of information in EHRs meant writing notes frequently, even when new notes were similar to previous notes. Participants described EHR documentation as a constant process consuming a considerable portion of the day. Patient load was estimated as ranging from an average of 10 to 15 patients per day. With that, participants estimated that documentation time averaged 30 to 45 minutes per patient. Participants described documentation as redundant, as they and their trainees were required to write notes for the same patients.

Table 1 summarizes the contributing work system factors for each task perceived as frequent, redundant, and difficult with some illustrative quotes from participants.

**Table 1:** Frequent, redundant, and difficult tasks identified by participants and derived contributing work system factors.

Task	Contributing work system factor(s)	Representative quotation
Chart review^a^	People: Extent of reliance on electronic records varied between participants Environment: Electronic chart was not accessible at bedsides Tools/technology: EHR^b^ did not push notifications of important changes Tasks: Patients with more status changes needed more frequent review Organization: Multifactor authentication was required before every EHR session	“Ideally, you would like to be able to harvest that information in the room with the patient by handheld device so that if memory fails and patients have questions, you can use that to help answer their questions. Mostly, I do that from memory now.”
Orders^c^	People: Preferences varied in when to start and when to submit orders Environment: Electronic ordering was not accessible at bedsides Tools/technology: Finding the right order form in the EHR was difficult Tasks: Orders depended on having the most up-to-date patient information Organization: All orders had to be made through the EHR	“There are multiple clicks to get to different boxes, lots of pop-ups that you have to go through...the computer system itself adds considerably to the amount of time that we take and takes away from our patient care”
Documentation^d^	People: Content of attendings’ notes depended on the content of their residents’ notes Environment: EHR was not accessible at bedsides Tools/technology: Authoring notes in the EHR sometimes involved copying forward text from older notes Tasks: These were sometimes based on a single encounter, and other times more longitudinal (eg, discharge summaries) Organization: Facility required a series of documentation and ordering steps before discharge	“I think documentation is by far the thing that takes us the longest— documentation for sure.”

^a^Chart review: going through patient information and history.

^b^EHR: electronic health record.

^c^Orders: services like lab tests and referral.

^d^Documentation: summarizing encounters, making or changing care plans, and adding to patient information.

### Impact of Tasks: Inefficient and Difficult Hospitalists’ Workflows

Participants characterized admit, discharge, and rounding workflows as difficult or inefficient.

#### Intake

The admit workflow was reported to be time-consuming:

It takes 1.5-2 hours to do an admission from start to finish,...entails chart review, seeing the patient, putting in orders, reviewing things, and doing the history and physical.

Difficulty of completing tasks seemingly increased as the workday progressed. Often, patients’ care was distributed across multiple health care systems. In those cases, admitting was described as involving retrieving both internal and external records. At best, external records were retrieved electronically (eg, from a health information exchange). Otherwise, retrieving outside records involved making telephone calls and reviewing scanned records. Some participants relied on residents:

I usually have learners helping take care of some other tasks but without learners sometimes it [compiling patients’ histories] just doesn’t happen.

#### Discharge

Most participants noted efforts to complete discharges by lunchtime:

...it’s usually like a 4-5 hour process. It’s challenging to discharge patients in the afternoon, because there’s just too much to get done. It’s cumbersome...

Due to documentation demands, participants described these workflows as redundant and time-consuming. Discharges involved data retrieval that depended on the length of the stay and much documentation. Several notes needed to be written, and among those notes, a large amount of information was duplicated:

...So discharge note, anticipated note, discharge instruction, discharge summary, medical reconciliation, pharmacy output...we can clump together to save time...

These characteristics related to admit and discharge workflows increased participants’ time in workrooms because access to their desktops were required to complete notes.

#### Rounds

Rounding was identified as an inefficient workflow. Participants reported seeing 10 to 18 patients during rounding. For each patient, participants documented history and physical notes in the EHR. Afterward, they duplicated the text on paper cards to support rounding. Otherwise, the information was not readily available. Printouts were wasteful because page counts averaged 6 pages per patient. Unlike computers and the EHR, paper notes fit in their pockets and were easily accessible for review and modification:

Bedside computers are not at the bedside. We don’t really have access to computers that work very well other than those in our team rooms.

**Figure 1:**
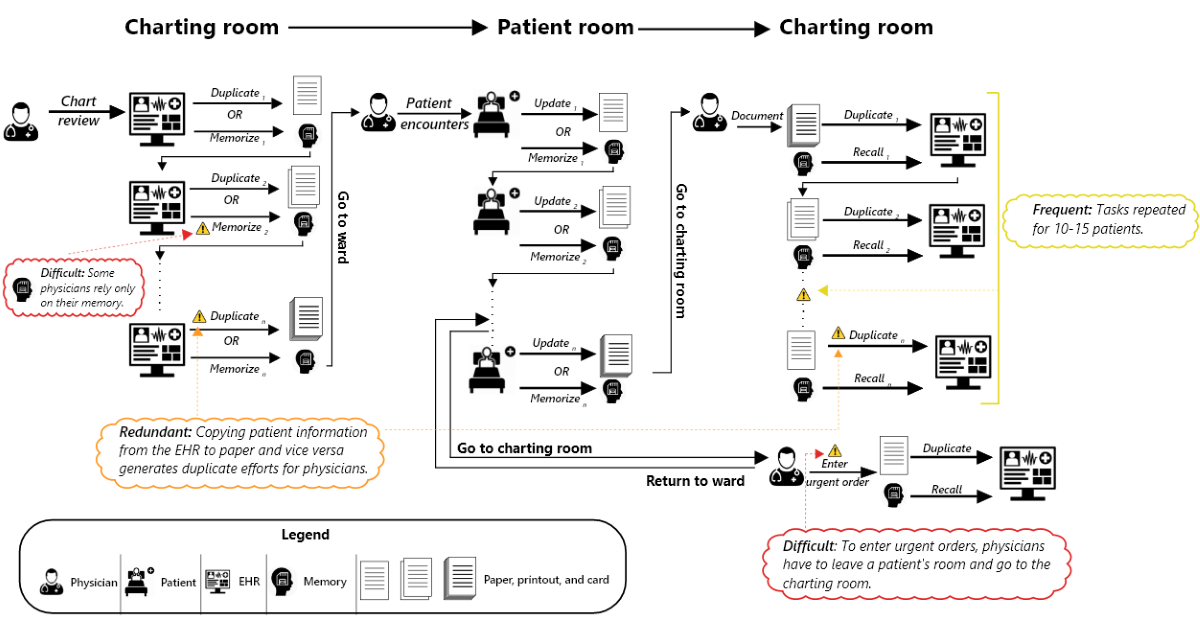
Snapshot of difficult and inefficient elements in hospitalists’ workflows. Hospitalists start in the charting room and conduct chart reviews for all patients they will visit. For each patient, hospitalists must duplicate information from the EHR on index cards or printouts to support review at bedside. After completing cards, hospitalists take all the cards to the ward where patients are located. Hospitalists find the appropriate card for each patient encounter and update the card with new patient information related to status, orders, and plans. Hospitalists move from one patient to the next, repeating those steps. After the last patient encounter, hospitalists go back to the charting room to enter the information from the cards into the EHR. This entire workflow is done multiple times a day. EHR: electronic health record.

Based on participant interviews, Figure 1 illustrates a snapshot of the participants’ description of difficult and inefficient workflows that stem from frequent, redundant, and difficult tasks.

### Participants’ Perspectives on Opportunities for Mobile App Interventions

Participants expected mobile technology to decrease task completion time; however, they noted that neither rapid access nor documentation of information was supported by current mobile apps. Usability issues were also noted, highlighting the misalignment between expected and actual functionality. One participant said:

I had an iPad for a while here when I was in the pain clinic, but I didn’t use it. I couldn’t do controlled substances refills on it, and that was all that I ordered in the pain clinic. So I turned it back in...

As a result, participants expressed that they viewed paper and their brains as “surrogate mobile devices.” Paper to-do lists were described as repositories of patient information and task trackers. Paper was perceived as more reliable than mobile technology. Alternatively, some participants described heavy reliance on their memory. As one participant noted, pointing to his head, “my technology is up here.”

When discussing ideas for mobile technology, participants prioritized portability, reduction in task time completion and task completion at bedside (Textbox 1). Three representative examples of useful mobile apps emerged. First, participants said a mobile device like an iPad would help patient-provider communication and entering orders at bedside. Second, participants described a note-taking app that had sharing features and stored nurses contact information. Lastly, participants proposed an app for electronic consents.

**Participant quotes describing potential mobile app solutions.**

**Patient-provider communication**

“...instead of telling patients, actually giving them a visual as you are rounding will make them help feel more involved in their own care.”

**Team communication**

“...[communication] breakdown occurs when we’re calling nurses...if we just had the correct number in the first place, we wouldn’t have to go through talking to multiple people.”

**eConsent**

“...when you go buy a coffee and doughnuts, you know how you can just sign on the iPad; having that same setup for consent may work well.”

## Discussion

### Summary

Provider-EHR interactions in inpatient care have contributed to increased workflow inefficiencies, reduced time for provider-patient interactions, and increased cognitive burden among hospitalists. Our findings provide a better understanding of the misalignment between hospitalists’ needs and expectations of available mobile technology and evidence of hospitalists’ cognitively intense workflows. In this section, we discuss our findings and implications for implementation of future mobile technology interventions for hospitalists.

### Influential Contextual Factors

Despite the need for mobile access to patient information, mobile technology was not widely adopted. Difficult and inefficient tasks were predominately related to provider-EHR interactions because access to EHRs was not consistent at bedside [33]. We associated hospitalists’ unmet needs with one or more of the following SEIPS factors: tasks, tools and technology, and environment (location). For example, participants had to travel across three floors to complete workflows and clinical tasks that required chart review, patient encounters, and documentation. These dynamics influenced the perceived difficulty, frequency, and redundancy among workflows and clinical tasks. Although mobile technology was available, usability issues related to existing mobile apps prevented their use, increasing participants’ reliance on index cards and printouts. Thus, contextual factors influenced the need for mobile technology, but misalignments of hospitalists’ expectations and mobile device functionality limited the adoption and use of existing mobile apps. This finding demonstrates the critical importance of integrating workflow analysis into the design process of mobile technology interventions; the result of this analysis identifies unmet needs and unintended consequences.

### Implications of Cognitive Workload and Burden

The three information-intensive tasks (chart review, ordering, and documentation) identified as frequent, redundant, and difficult were prone to an overreliance on hospitalists’ memory, including working memory [34]. A major contributor to this overreliance was the lack of mobile technology that supported chart review or order entry needed at bedside. Classifying tasks as frequent and redundant were easy for participants. These tasks were often described as inefficient and sources of hospitalists’ frustration. Identifying tasks as difficult caused participants to think of their tasks in a new way. Echoed throughout our data collection, tasks were not difficult due to hospitalists’ lack of knowledge or training to identify treatment plans, make clinical decisions, or perform clinical procedures. Rather, difficulty was defined and associated with the lack of support and access to usable technology required to review and enter information at bedside. According to existing cognition literature [35-37], these workflow aspects required participants to change location frequently, which increased the likelihood of interruptions and limited information recall (ie, cognitive slips and mistakes). This can be linked to incomplete documentation, communication breakdowns, and delays in care alluded to in participant interviews. Paper-based work-arounds were associated with processing orders and notes together in one sitting (ie, batch processing), not individually at the time of each decision. Batch processing has been associated with delayed team communication, delayed discharges, and shift limit violations [38]. Although the terms *cognitive burden* and *mental overload* were not specifically mentioned in interviews, these were clear outcomes for hospitalists based on our analysis. Cognitive burden can decrease resilience, situation awareness, and subsequently patient safety [36,39-41].

### Potential for Task-Specific Mobile Apps

Hospitalists thought task-specific apps would be most helpful. Their primary goals were to reduce inefficiencies or difficulties with orders, discharge, consent, and team communication. Hospitalists’ focus on individual tasks indicates a need to shift design goals of mobile apps that focused on granting access to the entire EHR via consistent user interfaces (eg, mobile version of EHR desktop interfaces). Participants stressed the need for task-specific apps that highlighted fast, focused technology interactions when away from the charting room. The design of mobile apps should be based on the objective and use of the paper or cards currently used for hospitalists’ mobile workflows, including quick review and documentation of prioritized patient information. For example, apps should present customized views of patient health status or trends. In addition, apps can support bedside order entry with *smart* templates that use automation or dictation to optimize data entry without keyboards. Based on our findings, mobile apps designed to support the iterative nature of hospitalists’ workflows or rounds by providing a means to review charts and document at bedside may reduce the need for batch processing before and after patient encounters. Thus, current workflows would be streamlined, decreasing the redundancy and difficulty illustrated in workflows characterized in our study.

There are several task-specific apps that are being trialed and should be monitored for success. Since the completion of our study, the VA’s Office of Connected Care is working to achieve greater understanding of provider preferences for mobile technology and task-specific apps. Providers currently have access to a variety of task-specific apps for mobile computing through the VA App Store. For example, the Image Viewing Solution is an app to access diagnostic-grade images. Annie App for Clinicians allows providers to assign disease-specific protocols to their patients. Several other task-specific apps are in development to meet VA providers’ needs.

### Limitations

This workflow analysis was limited by a relatively small sample in one health care facility. VA is the nation’s largest integrated health care system. Therefore, participant perspectives of hospitalists’ workflows and mobile technology may be broadly relevant to other health care systems. For example, initial deployment of mobile technology, without a variety of task-specific clinical apps readily available contributed to the low adoption of mobile technology [18,19]. By using informal definitions of frequent, redundant, and difficult, these concepts may have overlapped to some degree. We did not explore differences associated with career stage (eg, early, middle, and late). Our findings demonstrated the influence of contextual factors; future studies should further explore interactions between technology use, interruptions, and geographic cohorting across multiple facilities [35,42-44].

### Conclusion

Based on our results, there are opportunities for mobile technology to decrease the difficulty and increase the efficiency of hospitalists’ workflows. Mobile technology and task-specific mobile apps with enhanced usability have the potential to decrease overreliance on hospitalists’ memory and fragmentation of clinical tasks across locations that exist with current health IT and hospital environments. Task-specific apps that aim to reduce redundancies or excessive administrative work related to admissions, orders, and discharges were prioritized by hospitalists. Human factors engineering approaches are needed to identify hospitalists’ requirements for mobile technology to address issues with information management and recall during rounds. Extending beyond hardware features, a better understanding of direct and contextual factors of mobile information needs is required to develop mobile apps that can support hospitalists’ workflows. This will be influential in initial and sustained adoption of future mobile technology and apps.

## Appendix

Multimedia Appendix 1 Interview guide.

## Abbreviations

EHR: electronic health record
IT: information technology
SEIPS: Systems Engineering Initiative for Patient Safety
VA: Veterans Affairs

## References

[1] Saleem JJ, Russ AL, Neddo A, Blades PT, Doebbeling BN, Foresman BH (2011). Paper persistence, workarounds, and communication breakdowns in computerized consultation management. Int J Med Inform.

[2] Lowry SZ, Ramaiah M, Patterson ES, Brick D, Gurses AP, Ozok A, Simmons D, Gibbons MC (2014). Integrating Electronic Health Records Into Clinical Workflow: An Application of Human Factors Modeling Methods to Ambulatory Care.

[3] Russ AL, Saleem JJ, Justice CF, Woodward-Hagg H, Woodbridge PA, Doebbeling BN (2010). Electronic health information in use: characteristics that support employee workflow and patient care. Health Informatics J.

[4] Kim MO, Coiera E, Magrabi F (2017). Problems with health information technology and their effects on care delivery and patient outcomes: a systematic review. J Am Med Inform Assoc.

[5] Carayon P, Wetterneck TB, Rivera-Rodriguez AJ, Hundt AS, Hoonakker P, Holden R, Gurses AP (2014). Human factors systems approach to healthcare quality and patient safety. Appl Ergon.

[6] Erickson SM, Rockwern B, Koltov M, McLean RM, Medical Practice and Quality Committee of the American College of Physicians (2017). Putting patients first by reducing administrative tasks in health care: a position paper of the American College of Physicians. Ann Intern Med.

[7] Wachter RM, Goldman L (2016). Zero to 50,000 - The 20th Anniversary of the Hospitalist. N Engl J Med.

[8] Elliott DJ, Young RS, Brice J, Aguiar R, Kolm P (2014). Effect of hospitalist workload on the quality and efficiency of care. JAMA Intern Med.

[9] Salahuddin L, Ismail Z (2015). Classification of antecedents towards safety use of health information technology: a systematic review. Int J Med Inform.

[10] Weigl M, Müller A, Vincent C, Angerer P, Sevdalis N (2012). The association of workflow interruptions and hospital doctors' workload: a prospective observational study. BMJ Qual Saf.

[11] Sharma A, Lo V, Lapointe-Shaw L, Soong C, Wu PE, Wu RC (2017). A time-motion study of residents and medical students performing patient discharges from general internal medicine wards: a disjointed, interrupted process. Intern Emerg Med.

[12] Gurvich I, O’Leary KJ, Wang L, Van Mieghem JA (2020). Collaboration, interruptions, and changeover times: workflow model and empirical study of hospitalist charting. Manufacturing Serv Operations Manage.

[13] Baumann LA, Baker J, Elshaug AG (2018). The impact of electronic health record systems on clinical documentation times: a systematic review. Health Policy.

[14] Tsai CY, Pancoast P, Duguid M, Tsai C (2014). Hospital rounding--EHR's impact. Int J Health Care Qual Assur.

[15] Assis-Hassid S, Grosz BJ, Zimlichman E, Rozenblum R, Bates DW (2019). Assessing EHR use during hospital morning rounds: a multi-faceted study. PLoS One.

[16] Rao AS, Adam TJ, Gensinger R, Westra BL (2012). Study of the factors that promoted the implementation of electronic medical record on iPads at two emergency departments. AMIA Annu Symp Proc.

[17] Ventola CL (2014). Mobile devices and apps for health care professionals: uses and benefits. P T.

[18] Walsh C, Stetson P (2012). EHR on the move: resident physician perceptions of iPads and the clinical workflow. AMIA Annu Symp Proc.

[19] Horng S, Goss FR, Chen RS, Nathanson LA (2012). Prospective pilot study of a tablet computer in an Emergency Department. Int J Med Inform.

[20] O'Leary KJ, Liebovitz DM, Baker DW (2006). How hospitalists spend their time: insights on efficiency and safety. J Hosp Med.

[21] Tipping MD, Forth VE, O'Leary KJ, Malkenson DM, Magill DB, Englert K, Williams MV (2010). Where did the day go?--a time-motion study of hospitalists. J Hosp Med.

[22] Saleem JJ, Savoy A, Etherton G, Herout J (2018). Investigating the need for clinicians to use tablet computers with a newly envisioned electronic health record. Int J Med Inform.

[23] Holden RJ, Carayon P, Gurses AP, Hoonakker P, Hundt AS, Ozok AA, Rivera-Rodriguez AJ (2013). SEIPS 2.0: a human factors framework for studying and improving the work of healthcare professionals and patients. Ergonomics.

[24] Crandall B, Klein GA, Hoffman RR (2006). Working Minds: A Practitioner's Guide to Cognitive Task Analysis.

[25] Guest G, Bunce A, Johnson L (2016). How many interviews are enough?. Field Methods.

[26] Pantilat S (2006). What is a hospitalist?. The Hospitalist.

[27] Cowan MJ, Shapiro M, Hays RD, Afifi A, Vazirani S, Ward CR, Ettner SL (2006). The effect of a multidisciplinary hospitalist/physician and advanced practice nurse collaboration on hospital costs. J Nurs Adm.

[28] Morgeson FP, Rogelberg SG (2017). Job analysis methods. The SAGE encyclopedia of industrial and organizational psychology.

[29] Morgeson FP, Brannick MT, Levine EL (2019). Job and Work Analysis: Methods, Research, and Applications for Human Resource Management.

[30] Unertl KM, Weinger MB, Johnson KB, Lorenzi NM (2009). Describing and modeling workflow and information flow in chronic disease care. J Am Med Inform Assoc.

[31] Unertl KM, Novak LL, Johnson KB, Lorenzi NM (2010). Traversing the many paths of workflow research: developing a conceptual framework of workflow terminology through a systematic literature review. J Am Med Inform Assoc.

[32] Hamilton AB, Finley EP (2019). Qualitative methods in implementation research: an introduction. Psychiatry Res.

[33] Clynch N, Kellett J (2015). Medical documentation: part of the solution, or part of the problem? A narrative review of the literature on the time spent on and value of medical documentation. Int J Med Inform.

[34] Cowan N (2010). The magical mystery four: how is working memory capacity limited, and why?. Curr Dir Psychol Sci.

[35] Zheng K, Haftel HM, Hirschl RB, O'Reilly M, Hanauer DA (2010). Quantifying the impact of health IT implementations on clinical workflow: a new methodological perspective. J Am Med Inform Assoc.

[36] Radvansky GA, Krawietz SA, Tamplin AK (2011). Walking through doorways causes forgetting: further explorations. Q J Exp Psychol (Hove).

[37] Murre JMJ, Dros J (2015). Replication and analysis of Ebbinghaus' forgetting curve. PLoS One.

[38] Calderon AS, Blackmore CC, Williams BL, Chawla KP, Nelson-Peterson DL, Ingraham MD, Smith DL, Kaplan GS (2014). Transforming ward rounds through rounding-in-flow. J Grad Med Educ.

[39] Farrell LJ, Du S, Steege LM, Cartmill RS, Wiegmann DA, Wetterneck TB, Hoffmann AE, Endsley MR (2017). Understanding cognitive requirements for EHR design for primary care teams. Proc Int Symp Hum Factors Ergonomics Health Care.

[40] Belden JL, Patel J, Lowrance N, Plaisant C, Koopman R, Moore J, Johnson TR, Sonin J (2014). Inspired EHRs: Designing for Clinicians.

[41] Singh H, Giardina TD, Petersen LA, Smith MW, Paul LW, Dismukes K, Bhagwath G, Thomas EJ (2012). Exploring situational awareness in diagnostic errors in primary care. BMJ Qual Saf.

[42] Kara A, Flanagan ME, Gruber R, Lane KA, Bo N, Kroenke K, Weiner M (2020). A time motion study evaluating the impact of geographic cohorting of hospitalists. J Hosp Med.

[43] Lopetegui M, Yen PY, Lai AM, Embi PJ, Payne PRO (2012). Time Capture Tool (TimeCaT): development of a comprehensive application to support data capture for Time Motion Studies. AMIA Annu Symp Proc.

[44] Ballermann MA, Shaw NT, Mayes DC, Gibney RTN, Westbrook JI (2011). Validation of the Work Observation Method By Activity Timing (WOMBAT) method of conducting time-motion observations in critical care settings: an observational study. BMC Med Inform Decis Mak.

