# Supplementary material for: Clinician Perspectives on Unmet Needs for Mobile Technology Among Hospitalists: Workflow Analysis Based on Semistructured Interviews
Source: JMIR Hum Factors. 2022 Jan 4;9(1):e28783. doi: 10.2196/28783 (PMC8767475; doi:10.2196/28783)
Supplement: Multimedia Appendix 1 [file humanfactors_v9i1e28783_app1.docx]

# Appendix: Interview Guide

1. What would you say is/are your most FREQUENT task(s)? Please explain.
2. What would you say is/are your most REDUNDANT task(s)? Please explain.
3. What task(s) would you say is/are most DIFFICULT? Please explain.

[Then, for each task identified above, ask the following:]

1. Was this task mobile or performed away from your desk/office? If so, where was it performed?
2. What types of information do you need to complete this task?
3. What tools and technology do use to complete this task?
4. With whom do you communicate with to complete this task?
5. In what ways, if any, do organizational factors (for example: work schedules, work culture, management, and training) or policies affect your ability to complete this task? Using mobile apps?
6. How could mobile applications and technology help support this task?
